# Evaluating and modifying the PHDI for depression prevention: insights from NHANES 2005–2018

**DOI:** 10.3389/fnut.2025.1601129

**Published:** 2025-05-30

**Authors:** Jia-xin Tan, Qing-zhong Li, Yuan-xin Mo, Hong-ping Zhou, Liu Miao, Guo-tian Ruan, Teng Deng, Yi-zhen Gong

**Affiliations:** ^1^Department of Clinical Research, Guangxi Medical University Cancer Hospital, Nanning, Guangxi, China; ^2^Department of Neurosurgery, Guangxi Medical University Cancer Hospital, Nanning, Guangxi, China; ^3^Department of Respiratory Oncology, Guangxi Medical University Cancer Hospital, Nanning, China; ^4^Department of Anesthesia and Surgery Center, Wuming Hospital of Guangxi Medical University, Nanning, Guangxi, China; ^5^Department of Cardiology, Liuzhou People's Hospital, Affiliated of Guangxi Medical University, Liuzhou, Guangxi, China; ^6^The Key Laboratory of Coronary Atherosclerotic Disease Prevention and Treatment of Liuzhou, Liuzhou, China; ^7^Department of General Surgery, Beijing Friendship Hospital, Capital Medical University, Beijing, China

**Keywords:** EAT-Lancet diet, Planetary Health Diet Index, depression, machine learning, logistic regression, dietary

## Abstract

**Background:**

Depression is a significant focus in mental health research, emerging as a pressing public health concern globally. The Planetary Health Diet Index (PHDI), recently proposed by The Lancet to balance health and environmental sustainability, remains unclear in its role in preventing depression—our study aims to investigate this association and seeks to optimize this dietary index.

**Methods:**

A cross-sectional analysis was conducted using 2005–2018 NHANES data from 27,868 participants. Dietary quality was measured using PHDI-US, and depressive symptoms were assessed with the Patient Health Questionnaire-9 (PHQ-9; score ≥10 indicating depressive symptoms). Associations between dietary indices and depressive symptoms were analyzed using multivariable logistic regression and restricted cubic splines. Machine learning identified key PHDI-US components, leading to the recalibration of PHDI-US to create PHDI-Fruits.

**Findings:**

Except for the Dietary Inflammatory Index (DII), the PHDI-US, HEI-2020, AHEI, and MEDI all demonstrated protective effects against depression. However, the benefits of PHDI-US were weaker compared to the other indices, particularly among participants with high adherence. Among the components of PHDI-US, fruits had the most significant impact. After recalibrating the PHDI-Fruits component, its ability to reduce depression incidence improved substantially, surpassing that of the other dietary indices.

**Interpretation:**

Optimizing the preventive effect of PHDI on depression, the recalibrated PHDI-Fruits significantly enhances its ability to prevent depression, effectively improving the applicability of PHDI in populations affected by depression.

## 1 Background

Depression is a prevalent and complex psychiatric disorder that significantly impacts global mental health, functional capacity, and quality of life, while imposing a substantial socioeconomic burden (1). Between 1990 and 2017, the global prevalence of depression increased by nearly 50% (2, 3) In the United States, the prevalence among adults ranges from 7.2% to 9.2%, with no signs of reduction in its public health burden (4). The etiology of depression is multifactorial, involving genetic susceptibility, environmental exposures, and modifiable lifestyle factors. Among these, dietary patterns and physical activity have emerged as critical modulators in the pathophysiology of depression (5). Advances in nutritional psychiatry highlight the role of dietary quality in preventing and managing depressive symptoms. Diet, while essential for physical health, also profoundly impacts mental health and environmental sustainability (6–8). In 2019, the EAT-Lancet Commission introduced the Planetary Health Diet (PHD), a predominantly plant-based model aimed at alleviating diet-related non-communicable diseases (NCDs) and reducing the ecological footprint of food production (9–12). This model emphasizes nutrient-dense, plant-based foods such as fruits, vegetables, whole grains, nuts, and legumes, while limiting red meat and ultra-processed food consumption to achieve health and sustainability goals (13). The U.S. Planetary Health Diet Index (PHDI-US) was developed to assess adherence to these guidelines in the context of American dietary patterns. In addition to promoting plant-based diets and limiting energy-dense foods, it aims to reduce chronic disease prevalence (14). Other validated dietary indices, such as the Alternative Healthy Eating Index (AHEI), Dietary Inflammatory Index (DII), Healthy Eating Index 2020 (HEI-2020), and Mediterranean Diet Score (MEDI), have also been widely used to evaluate dietary quality and its associations with health outcomes, including mental health (15–18). These indices share common features, such as promoting anti-inflammatory and nutrient-rich foods like fruits, vegetables, and whole grains, while discouraging consumption of refined sugars, saturated fats, and processed foods linked to systemic inflammation and neuropsychiatric disorders (19–23). However, while PHDI-US is recognized as a framework for improving dietary quality and sustainability, its relationship with mental health outcomes, particularly depression, remains underexplored. Moreover, whether PHDI-US offers advantages over other dietary indices in mitigating depressive symptoms is unclear. Understanding how PHDI-US and other indices influence depression risk, evaluating its preventive potential, and optimizing its component weighting are critical for guiding evidence-based dietary interventions tailored to mental health needs.

## 2 Objective

This study addressed these gaps using cross-sectional data from the 2005 to 2018 National Health and Nutrition Examination Survey (NHANES), a nationally representative survey with rigorous protocols to ensure data reliability and validity. Depressive symptoms were assessed using the Patient Health Questionnaire-9 (PHQ-9), with scores ≥10 indicating clinically significant symptoms. The primary objectives were to evaluate the association between PHDI-US and depressive symptoms, compare its predictive validity with other dietary indices (AHEI, DII, HEI-2020, and MEDI), and optimize PHDI-US by identifying the contributions of its components to depressive symptom prevention.

## 3 Methods

### 3.1 Study population and ethics

**Figure 1:**
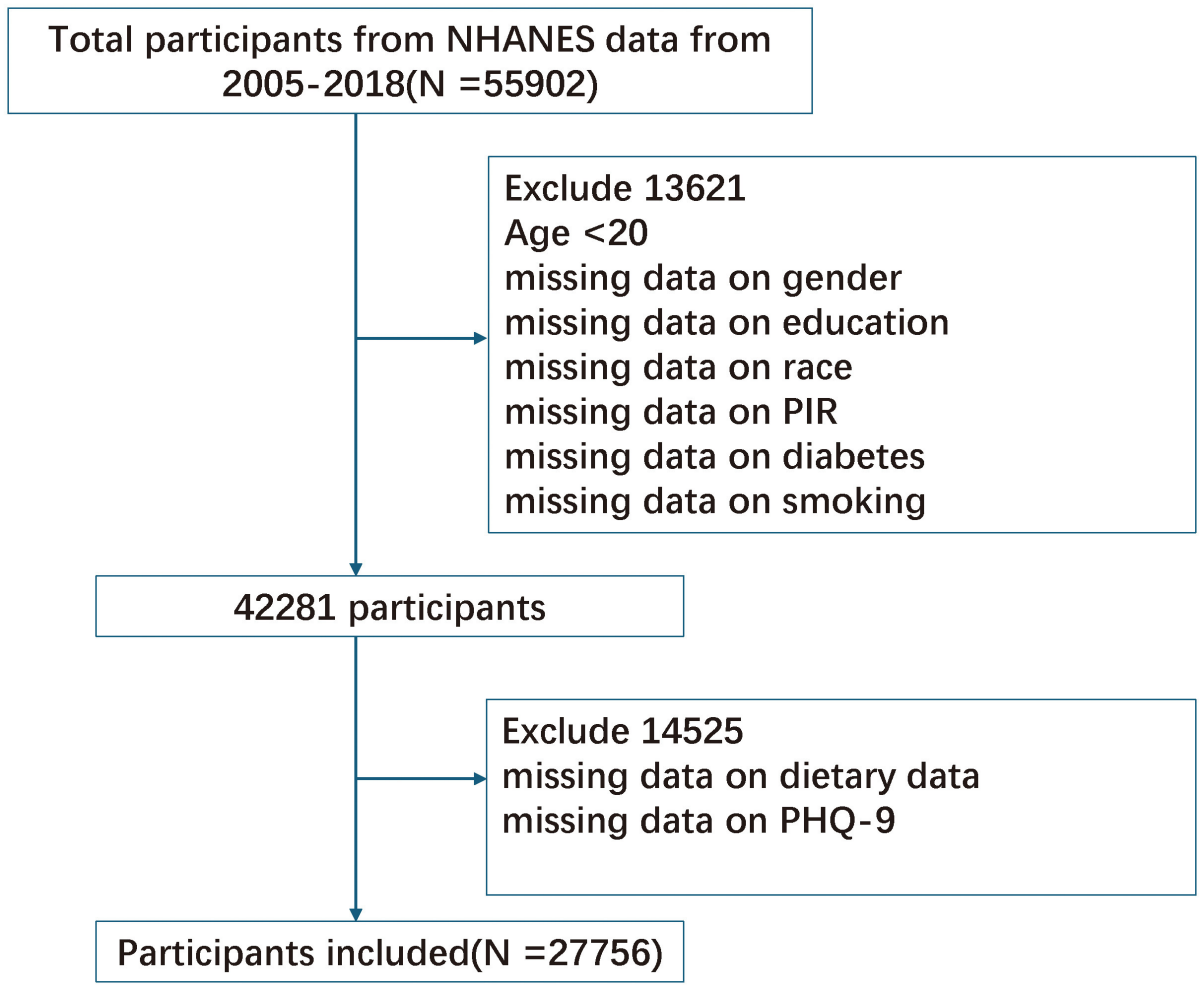
Flow chart of participants selection. NHANES, National Health and Nutrition Examination Survey.

This study used data from the National Health and Nutrition Examination Survey (NHANES), a health and nutrition survey conducted by the National Center for Health Statistics (NCHS). NHANES is a nationally representative survey designed to evaluate the health and nutritional status of the U.S. population. The data are publicly available and can be accessed from the NCHS website (https://www.cdc.gov/nchs/nhanes/index.htm). We analyzed cross-sectional data from seven NHANES cycles (2005–2018). Initially, 55,902 participants were included. Exclusion criteria were: (1) age below 20 years; (2) missing Patient Health Questionnaire-9 (PHQ-9) depression assessment data; (3) missing dietary data required for dietary index calculations; and (4) missing key covariates. After exclusions, 27,756 participants were included in the final analysis. The selection process is shown in Figure 1. The NHANES study protocol was approved by the NCHS Research Ethics Review Board. Written informed consent was obtained from all participants. The study was conducted following the principles of the Declaration of Helsinki.

### 3.2 Dietary assessment

Dietary intake information for NHANES participants was collected through computer-assisted, face-to-face interviews conducted in mobile examination centers (24). Data from the first-day 24-h dietary recall were analyzed for this study. A single 24-h dietary recall is considered a valid method for estimating population-level mean dietary intake (25). NHANES 2005–2018 data were used to assess total food and beverage intake (excluding water) and nutrient consumption for each participant. The USDA Food Patterns Equivalents Database (FPED) for NHANES 2005–2018 was used to calculate Food Patterns Equivalents (FPE) for each participant [http://www.ars.usda.gov]. FPE reflects individual food and beverage intake, categorized into 37 food pattern components. These components are also used to calculate scores for the Healthy Eating Index (HEI) [http://www.ars.usda.gov]. The U.S. Planetary Health Diet Index (PHDI-US) was constructed and scored based on the original PHDI framework. PHDI-US consists of 16 components, with a maximum possible score of 150 points. Higher PHDI-US scores indicate greater adherence to the dietary recommendations of the Planetary Health Diet (10). This index has undergone preliminary validation in the U.S. population and has been refined to better align with these dietary recommendations (14). The *DietaryIndex* is a standardized, user-friendly, and validated informatics tool designed for dietary index calculations. Validation files, source code, and detailed tutorials are publicly available on GitHub (https://github.com/jamesjiadazhan/dietaryindex) (26). This R package was utilized to compute the PHDI-US, Dietary Inflammatory Index (DII), Alternative Healthy Eating Index (AHEI), Healthy Eating Index 2015 (HEI-2015), and Healthy Eating Index 2020 (HEI-2020).

### 3.3 Outcome definition

Depressive symptoms were assessed using the Patient Health Questionnaire-9 (PHQ-9), a validated self-report instrument for screening depression. Participants responded to nine items reflecting the frequency of depressive symptoms over the past 2 weeks, yielding a total score ranging from 0 to 27. A score of ≥10 was used to define the presence of depressive symptoms, a threshold commonly employed in epidemiological studies due to its high sensitivity and specificity for detecting moderate to severe depression (27). Although the PHQ-9 is a self-reported measure and may be subject to recall bias, it has been extensively validated and is considered reliable for assessing depressive symptoms in population studies.

### 3.4 Covariates

Based on previous research and biological correlates, we included several potential confounders associated with depressive symptoms in our analysis. Demographic characteristics, such as age, sex, race/ethnicity, education, and smoking status, were collected through standardized questionnaires and face-to-face interviews. Physical examinations and laboratory tests were conducted by trained health professionals at MEC using standardized protocols. Race/ethnicity was categorized into five groups: non-Hispanic White, non-Hispanic Black, other Hispanic, Mexican American, and other races. Educational attainment was classified into three levels: below high school, high school graduate, and high school education. Smoking status was defined as having smoked at least 100 cigarettes in a lifetime, regardless of current smoking habits. Body Mass Index (BMI) was calculated by dividing weight (kg) by height squared (m^2^) to assess overweight/obesity status. Overweight was defined as a BMI between 25 and 30, while obesity was defined as a BMI >30. Systolic and diastolic blood pressure (SBP/DBP) were measured three times by a trained professional, with 30-s intervals between each measurement. The average of these readings was used to determine participants' blood pressure levels. Hypertension was included as a potential confounding factor and was defined by the following criteria: (1) mean systolic blood pressure ≥140 mmHg; (2) mean diastolic blood pressure ≥90 mmHg; (3) self-reported hypertension diagnosed by a physician; or (4) current use of antihypertensive medication. To analyze the impact of changes in dietary culture on the results, we conducted subgroup analyses according to the NHANES survey period (2005–2010 and 2011–2018). Total energy intake was not included as a covariate in our models due to its complex relationship with dietary patterns and potential multicollinearity issues.

### 3.5 Statistical analysis

All statistical analyses accounted for the complex, multistage probability sampling design of NHANES, including sample weights, stratification, and clustering, to ensure that the estimates were representative of the characteristics and distribution of the U.S. adult population. Analyses were performed using R software version 4.1.6 (The R Foundation, Vienna, Austria) and the “survey” package was utilized to appropriately handle the complex survey data. A two-sided *p*-value of < 0.05 was considered statistically significant. Continuous variables were presented as weighted means ± standard deviations (SD), while categorical variables were expressed as weighted frequencies and percentages. Comparisons between continuous variables were conducted using weighted Student's t-tests, whereas categorical variables were analyzed using the Rao-Scott chi-squared test. Participants were divided into quartiles based on their PHDI-US scores (Q1 representing the lowest quartile and Q4 the highest), with the Q1 group serving as the reference. To estimate the odds ratios (ORs) and 95% confidence intervals (CIs) for the association between PHDI-US and depressive symptoms, multivariable logistic regression models were constructed at three levels of adjustment. In Model 1, no covariates were adjusted. Model 2 included adjustments for age and sex, while Model 3 further adjusted for age, sex, race/ethnicity, poverty-to-income ratio (PIR), education level, body mass index (BMI), smoking status, and hypertension. These covariates were selected based on their potential influence on diet and depressive symptoms to address confounding factors and ensure a more robust estimation of the association.

In addition, restricted cubic spline (RCS) regression models were applied, with knots set at the 10th, 50th, and 90th percentiles of PHDI-US scores, to explore potential non-linear associations between PHDI-US and depressive symptoms. This method provides greater flexibility in modeling complex relationships. To evaluate potential effect modification, univariate subgroup analyses were conducted based on variables such as age, sex, BMI, education level, race/ethnicity, smoking status, income, hypertension, and diabetes. Interaction terms were included in the models to determine significant interactions between variables. Finally, to identify dietary elements most strongly associated with reduced depression risk, advanced machine learning methods, including random forest and feature importance analysis, were employed to evaluate the 16 food components of the PHDI-US. After identifying the most critical features for the model, the original PHDI was recalibrated based on these key features. Subsequently, logistic regression and linear regression models were used to compare the recalibrated PHDI with the original PHDI as well as with the other four dietary indices (AHEI, DII, HEI-2020, and MEDI). These comparisons assessed the differences in performance for reducing the risk of depressive symptoms and the linear relationship with depression scores. Although this study reveals a significant association between of this study, the results presented only reflect an association between diet and depression, rather than a causal relationship.

## 4 Results

### 4.1 Participant characteristics and inclusion/exclusion criteria

A total of 27,756 participants were included in the final analysis, which, after weighting and nesting, represents ~170 million people in the United States. Participants were stratified based on the presence of depressive symptoms (PHQ-9 ≥ 10). Among participants with depression, 52.18% were female, and 45.03% were young adults (aged 20–45 years) (Table 1). In the female subgroup, the proportion of participants with depressive symptoms was significantly higher than that of those without depressive symptoms (52.18% vs. 42.92%). A t-test or chi-square test was used to assess baseline differences between the MASLD group and the control group. The results showed significant differences in age, gender, race, education level, income, smoking, diabetes, hypertension, and BMI (*P* < 0.01). The PHDI score in the depressive symptom group was significantly lower than that in the control group (*P* < 0.01).

**Table 1:** Baseline characteristics of participants were stratified by presence of depressive symptoms.

**Characteristics**	**No depressive symptoms**	**With depressive symptoms**	***p*-value**
**PHDI-US**	64.48 (14.61)	61.60 (14.49)	< 0.001
**Gender**	–	–	< 0.001
Female	69,176,994 (42.92%)	6,617,850 (52.18%)	–
Male	91,997,647 (57.08%)	6,065,313 (47.82%)	–
**Age**	–	–	< 0.001
20–45	76,442,705 (47.43%)	5,711,281 (45.03%)	–
45–60	42,435,871 (26.33%)	4,283,738 (33.77%)	–
60–75	30,741,785 (19.07%)	2,126,032 (16.76%)	–
>75	11,554,281 (7.17%)	562,112 (4.43%)	–
**BMI**	–	–	< 0.001
< 18.5	2,410,727 (1.50%)	280,866 (2.21%)	–
18.5–25	45,875,007 (28.46%)	3,039,127 (23.96%)	–
25–30	53,043,243 (32.91%)	3,250,046 (25.62%)	–
>30	59,845,665 (37.13%)	6,113,123 (48.20%)	–
**Race**	–	–	< 0.001
Mexican American	12,659,695 (7.85%)	964,192 (7.60%)	–
Other Hispanic	7,802,009 (4.84%)	889,052 (7.01%)	–
Non-Hispanic White	113,556,207 (70.46%)	8,324,658 (65.64%)	–
Non-Hispanic Black	16,157,107 (10.02%)	1,615,782 (12.74%)	–
Other Race - Including Multi-Racial	10,999,624 (6.82%)	889,478 (7.01%)	–
**PIR**	–	–	< 0.001
Below poverty threshold	50,053,270 (31.06%)	7,236,854 (57.06%)	–
Above poverty threshold	111,121,371 (68.94%)	5,446,309 (42.94%)	–
**Education**	–	–	< 0.001
Below high school	21,771,112 (13.51%)	2,986,904 (23.55%)	–
High school	36,905,155 (22.90%)	3,504,345 (27.63%)	–
Above high school	102,498,374 (63.59%)	6,191,913 (48.82%)	–
**Smoking**	–	–	< 0.001
No	90,634,962 (56.23%)	4,951,908 (39.04%)	–
Yes	70,539,679 (43.77%)	7,731,255 (60.96%)	–
**Hypertension**	–	–	< 0.001
No	111,222,783 (69.01%)	7,185,002 (56.65%)	–
Yes	49,951,858 (30.99%)	5,498,161 (43.35%)	–
**DM**	–	–	< 0.001
No	139,701,438 (86.68%)	9,969,498 (78.60%)	–
Yes	21,473,203 (13.32%)	2,713,665 (21.40%)	–

Data are expressed as mean ± stand error or frequency (percentage).

PIR, Ratio of family income to poverty; BMI, body mass index; DM, Diabetes Mellitus.

### 4.2 Association of PHDI/AHEI/DII/HEI/MEDI with depression

**Figure 2:**
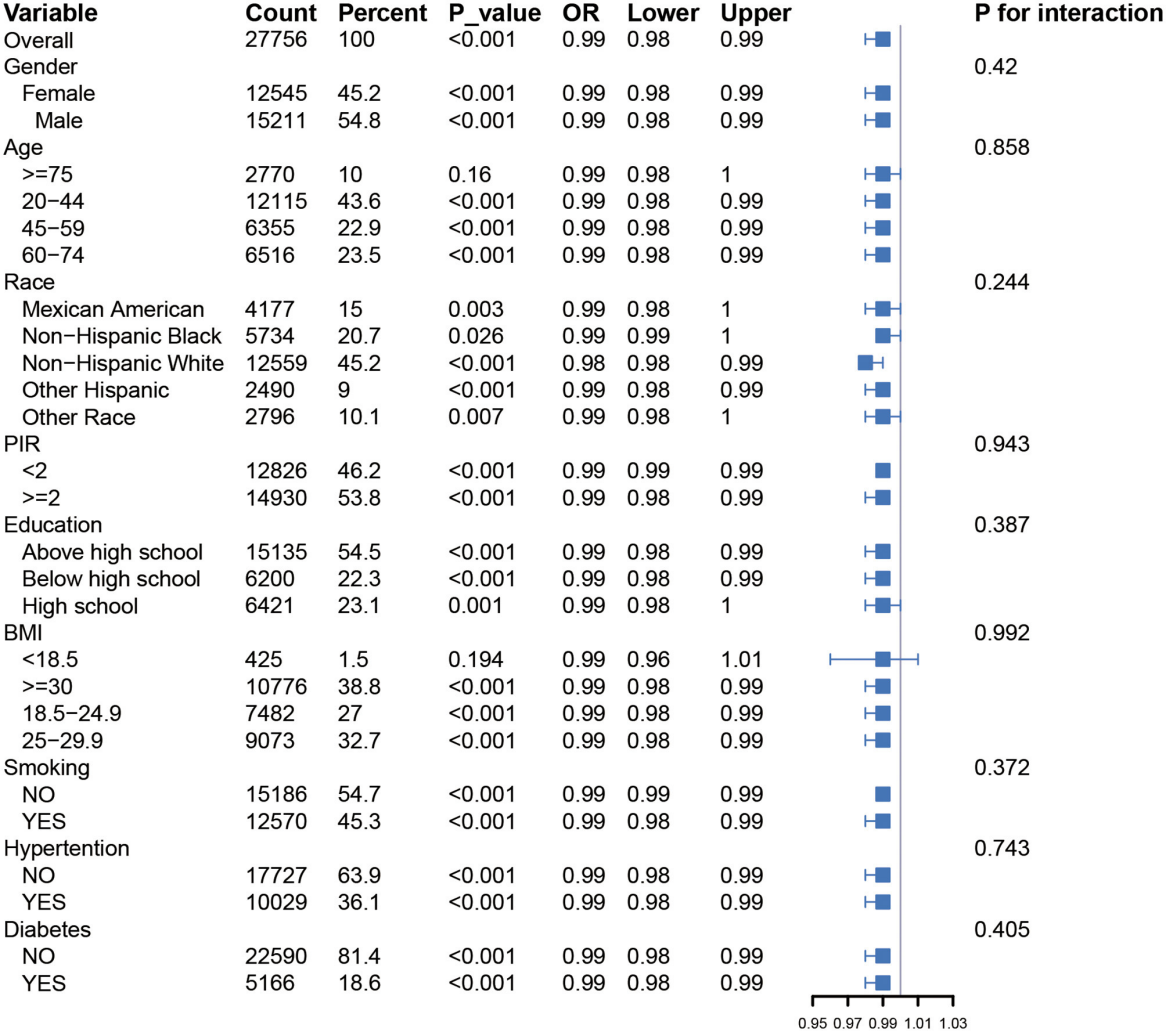
Forest plot showing univariate GLM regression analysis of Planetary Health Diet Index (PHDI) and depression prevalence across demographic and clinical subgroups.

**Figure 3:**
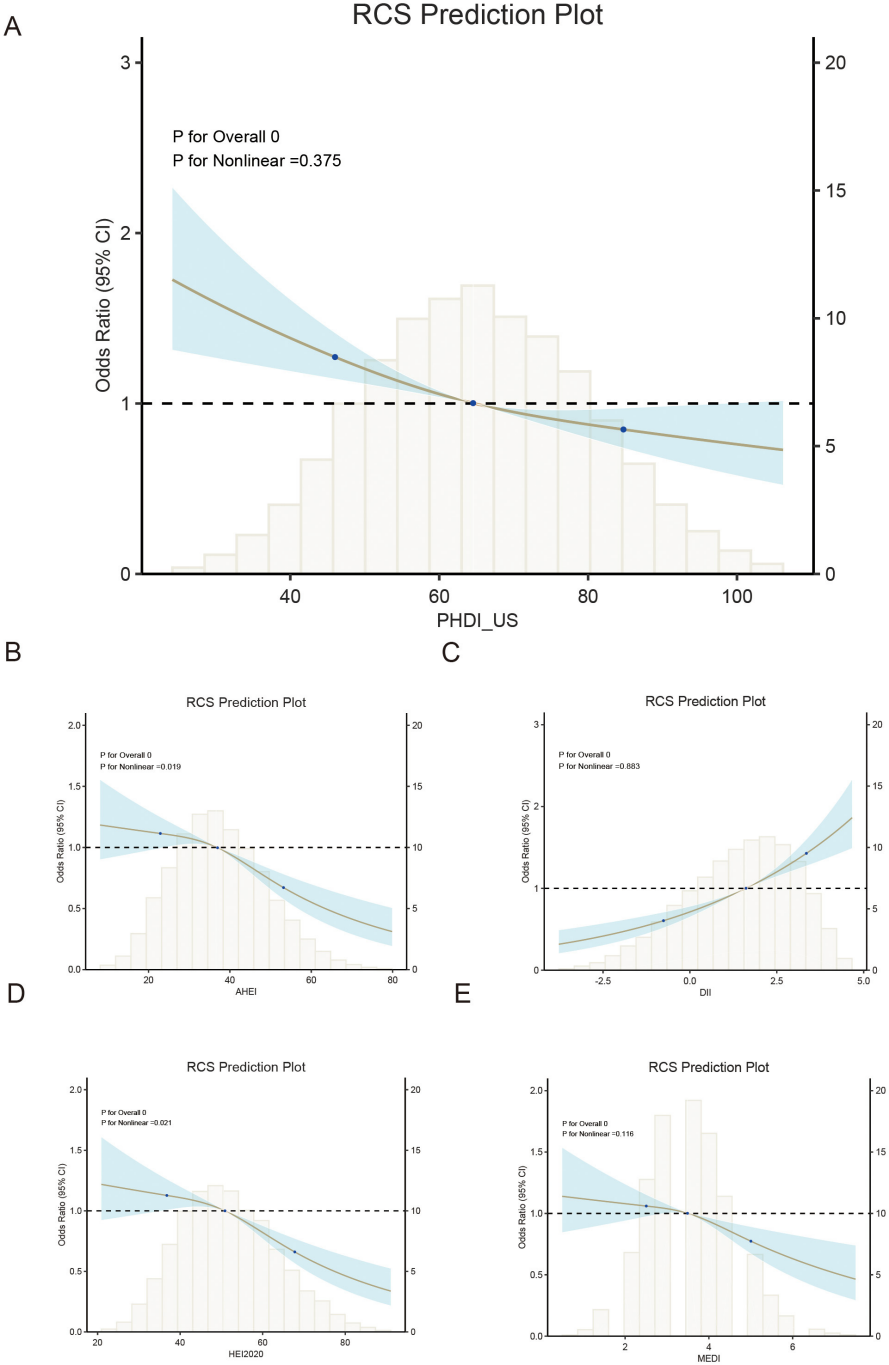
Dose-response relationship between dietary indices and depression risk using restricted cubic splines (RCS).

Subgroup analyses revealed a consistent inverse association between PHDI and depressive symptoms across all subgroups, including gender, age, BMI, race, poverty-income ratio (PIR), education level, smoking status, hypertension, and diabetes. No significant interactions were observed across these subgroups (*P* > 0.01). Detailed information is available in Figure 2. The associations of PHDI, AHEI, DII, HEI, and MEDI with depressive symptoms are summarized in Table 2. After full adjustment for covariates (Model 3), PHDI was significantly associated with a reduced likelihood of depressive symptoms. Participants in the highest quartile (Q4) of PHDI adherence had an odds ratio (OR) of 0.72 (95% CI: 0.62–0.83, *p* < 0.01) compared to those in the lowest quartile (Q1). This inverse relationship was consistent across quartiles (*p* for trend < 0.01). Among dietary indices, AHEI demonstrated the strongest association in the fourth quartile, with an OR of 0.67 (95% CI: 0.57–0.79, *p* < 0.01), indicating that higher adherence to AHEI was particularly effective in alleviating depressive symptoms. HEI also showed a strong association, with a Q4 OR of 0.63 (95% CI: 0.54–0.74, *p* for trend < 0.01). The association for MEDI was comparatively weaker, with an OR of 0.79 (95% CI: 0.69–0.91, *p* for trend < 0.01). In contrast, DII was positively associated with depressive symptoms. Participants in the highest quartile of DII adherence had an OR of 1.39 (95% CI: 1.65–2.26, *p* < 0.01), indicating that an inflammatory dietary pattern increased the risk of depressive symptoms. Restricted cubic spline (RCS) analyses for these five dietary indices demonstrated that higher levels of dietary adherence were associated with significantly reduced depressive risk. AHEI and HEI-2020 exhibited the most pronounced benefits at higher adherence levels. However, the PHDI curve showed a limited decline in depressive risk at higher adherence levels, with a wider confidence interval, suggesting its protective effect on mental health was less pronounced compared to other healthy dietary patterns. More details can be seen in Figure 3.

**Table 2:** Associations between dietary quality indices and depressive symptoms by quartiles.

**Variables**	**Model 1 [OR (95%CI)]**	***p*-value**	**Model 2 [OR (95%CI)]**	***p*-value**	**Model 3 [OR (95%CI)]**	***p*-value**
**PHDI**						
Quartile 1	Reference	–	Reference	–	Reference	–
Quartile 2	0.80 (0.70–0.91)	< 0.01	0.80 (0.70–0.91)	< 0.01	0.88 (0.77–1.01)	0.06
Quartile 3	0.74 (0.65–0.85)	< 0.01	0.76 (0.66–0.87)	< 0.01	0.84 (0.73–0.96)	0.01
Quartile 4	0.61 (0.53–0.71)	< 0.01	0.62 (0.54–0.72)	< 0.01	0.72 (0.62–0.83)	< 0.01
*p* for trend	–	< 0.01	–	< 0.01	–	< 0.01
**AHEI**						
Quartile 1	Reference	–	Reference	–	Reference	–
Quartile 2	0.80 (0.70–0.90)	< 0.01	0.80 (0.71–0.90)	< 0.01	0.91 (0.81–1.04)	0.17
Quartile 3	0.58 (0.51–0.66)	< 0.01	0.58 (0.51–0.66)	< 0.01	0.77 (0.67–0.89)	< 0.01
Quartile 4	0.40 (0.34–0.46)	< 0.01	0.40 (0.34–0.46)	< 0.01	0.67 (0.57–0.79)	< 0.01
*p* for trend	–	< 0.01	–	< 0.01	–	< 0.01
**DII**						
Quartile 1	Reference	–	Reference	–	Reference	–
Quartile 2	1.55 (1.32–1.83)	< 0.01	1.54 (1.30–1.81)	< 0.01	1.32 (1.12–1.56)	0.10
Quartile 3	2.05 (1.75–2.40)	< 0.01	2.01 (1.72–2.35)	< 0.01	1.59 (1.35–1.87)	< 0.01
Quartile 4	2.95 (2.54–3.44)	< 0.01	2.87 (2.47–3.34)	< 0.01	1.93 (1.65–2.26)	< 0.01
*p* for trend	–	< 0.01	–	< 0.01	–	< 0.01
**HEI**						
Quartile 1	Reference	–	Reference	–	Reference	–
Quartile 2	0.81 (0.71–0.91)	< 0.01	0.81 (0.71–0.92)	< 0.01	0.90 (0.79–1.03)	0.10
Quartile 3	0.61 (0.53–0.69)	< 0.01	0.61 (0.53–0.69)	< 0.01	0.78 (0.68–0.90)	< 0.01
Quartile 4	0.43 (0.37–0.50)	< 0.01	0.43 (0.37–0.50)	< 0.01	0.63 (0.54–0.74)	< 0.01
*p* for trend	–	< 0.01	–	< 0.01	–	< 0.01
**MEDI**						
Quartile 1	Reference	–	Reference	–	Reference	–
Quartile 2	0.83 (0.73–0.94)	0.12	0.84 (0.73–0.95)	< 0.01	0.89 (0.78–1.02)	0.08
Quartile 3	0.73 (0.63–0.85)	< 0.01	0.74 (0.64–0.86)	< 0.01	0.84 (0.73–0.98)	0.03
Quartile 4	0.59 (0.52–0.68)	< 0.01	0.60 (0.53–0.69)	< 0.01	0.79 (0.69–0.91)	< 0.01
*p* for trend	–	< 0.01	–	< 0.01	–	< 0.01

Model 1, No covariates were adjusted; Model 2, Adjusted for gender and age; Model 3, Adjusted for age, gender, race, PIR, education, BMI, smoking, and hypertension.

PHDI, Planetary Health Diet Index; AHEI, Alternative Healthy Eating Index; DII, Dietary Inflammatory Index; HEI, Healthy Eating Index. MEDI, Mediterranean Diet Index; OR, Odds Ratio; CI, Confidence Interval.

### 4.3 Recalibration of PHDI by enhancing the weight of fruit components to improve its association with depressive symptoms

**Figure 4:**
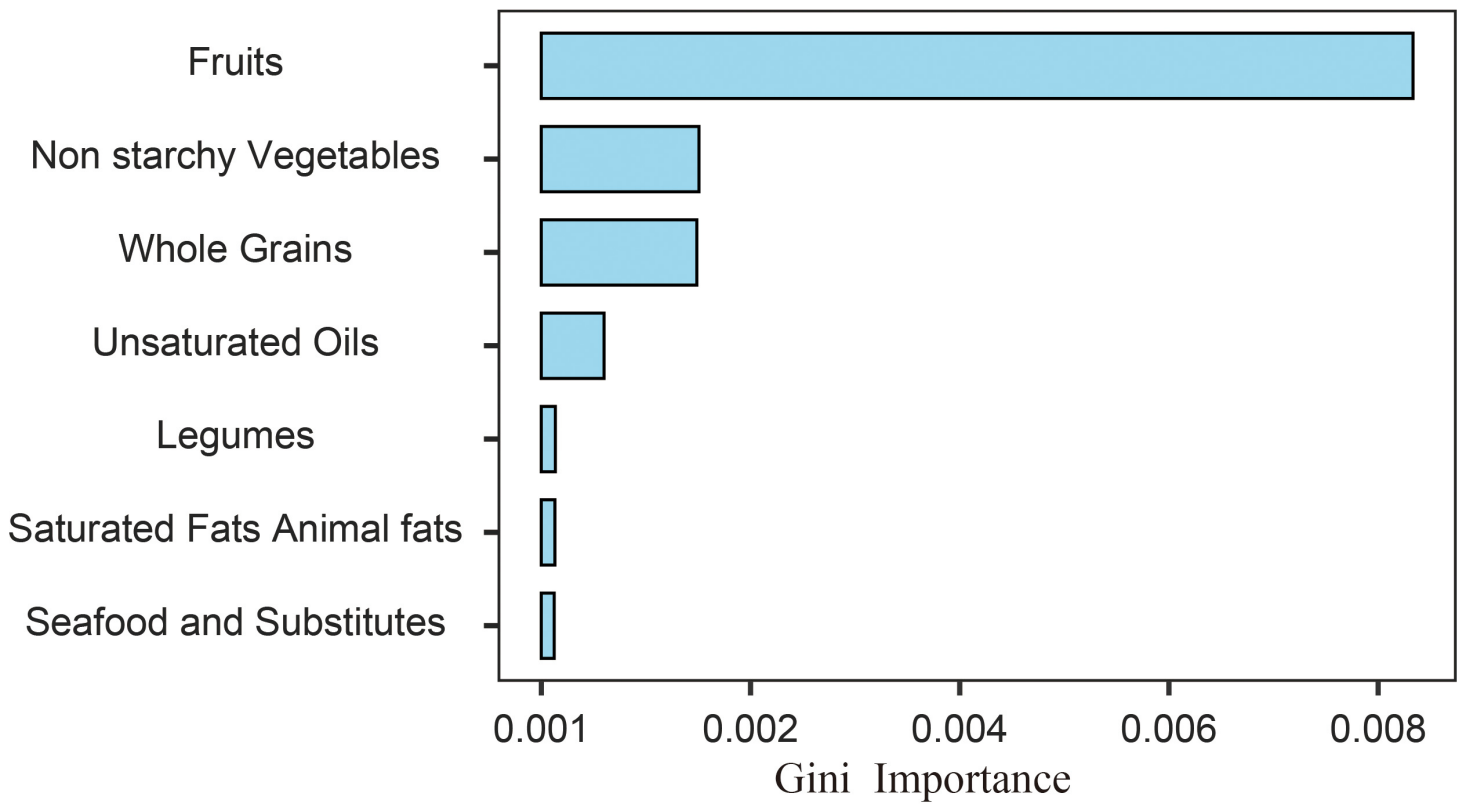
Feature importance ranking of PHDI components derived from random forest permutation analysis for predicting depressive symptoms.

To address the comparatively lower theoretical effectiveness of PHDI in preventing depressive symptoms compared to other dietary indices, a random forest algorithm was employed to identify the most important components among the 16 dietary components of PHDI. The results of the feature importance analysis (Figure 4) demonstrated that “Fruits” was the most influential component in reducing the risk of depressive symptoms. Based on this finding, the PHDI was recalibrated by incorporating a weighted adjustment with the fruit component, resulting in the modified index termed PHDI-Fruits.

**Figure 5:**
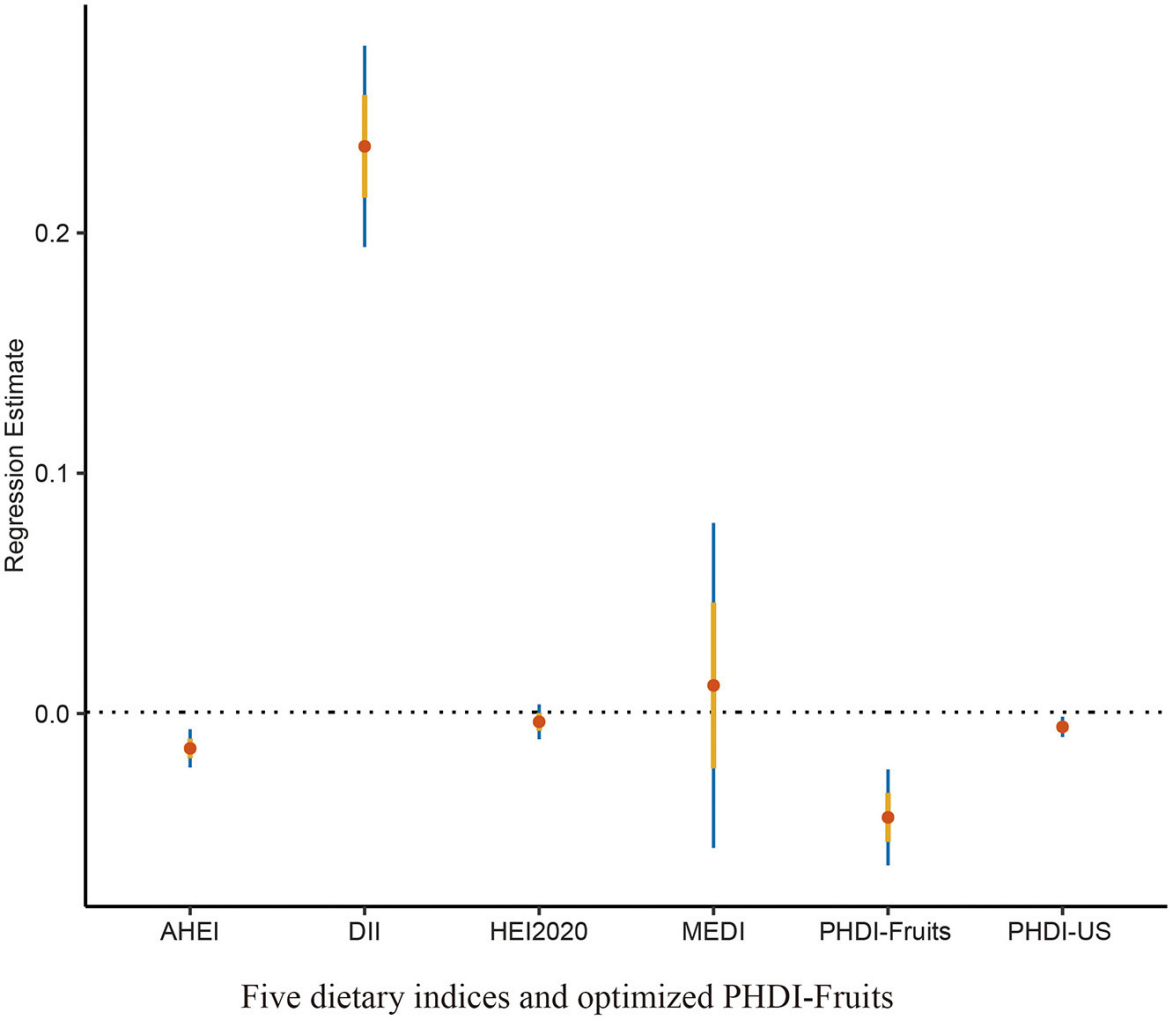
Linear regression coefficients for dietary indices in relation to depression risk.

**Figure 6:**
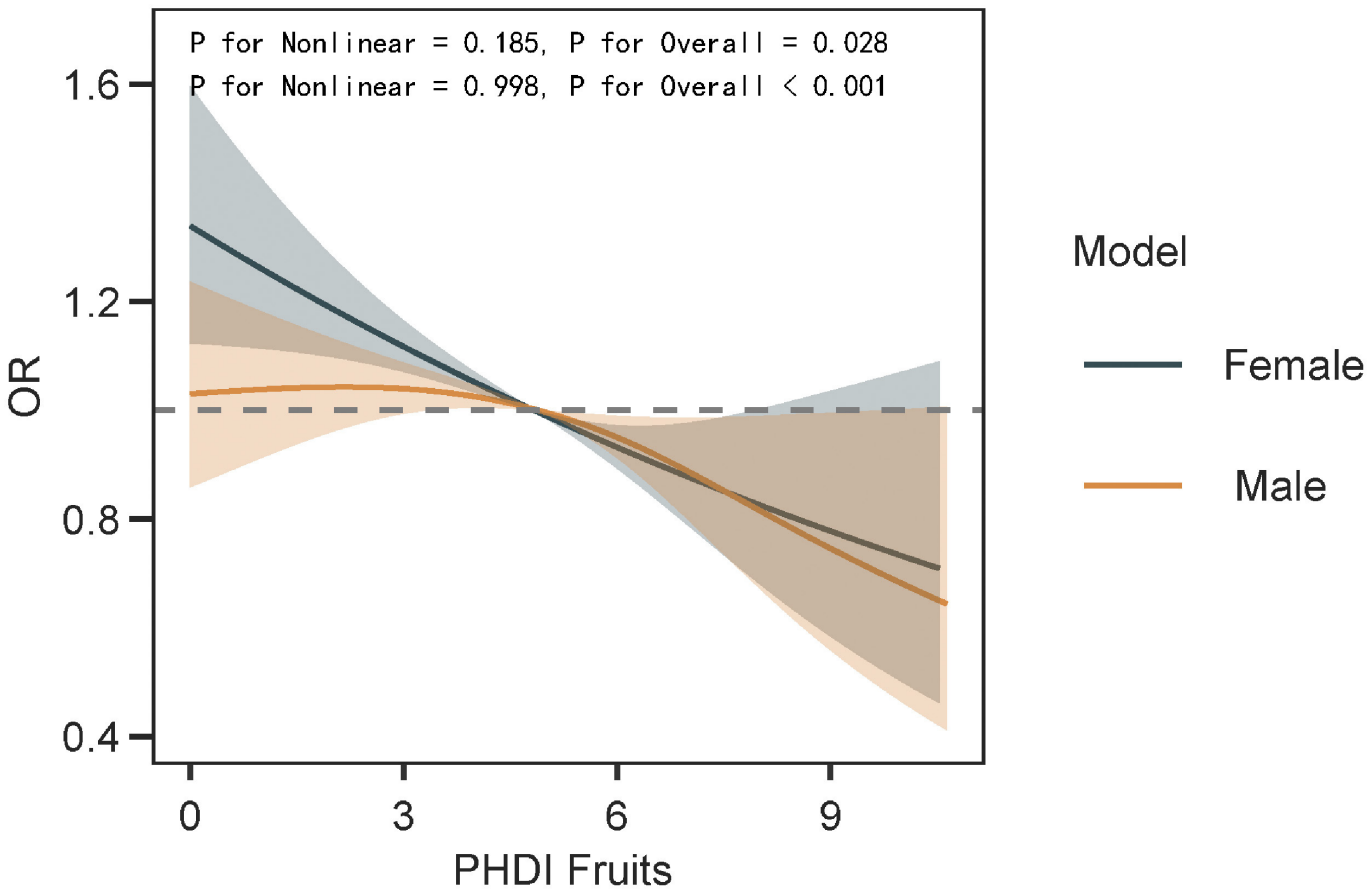
The dose-response relationship between PHDI-Fruits and depression risk in gender subgroups was examined using multivariable restricted cubic splines (RCS).

**Figure 7:**
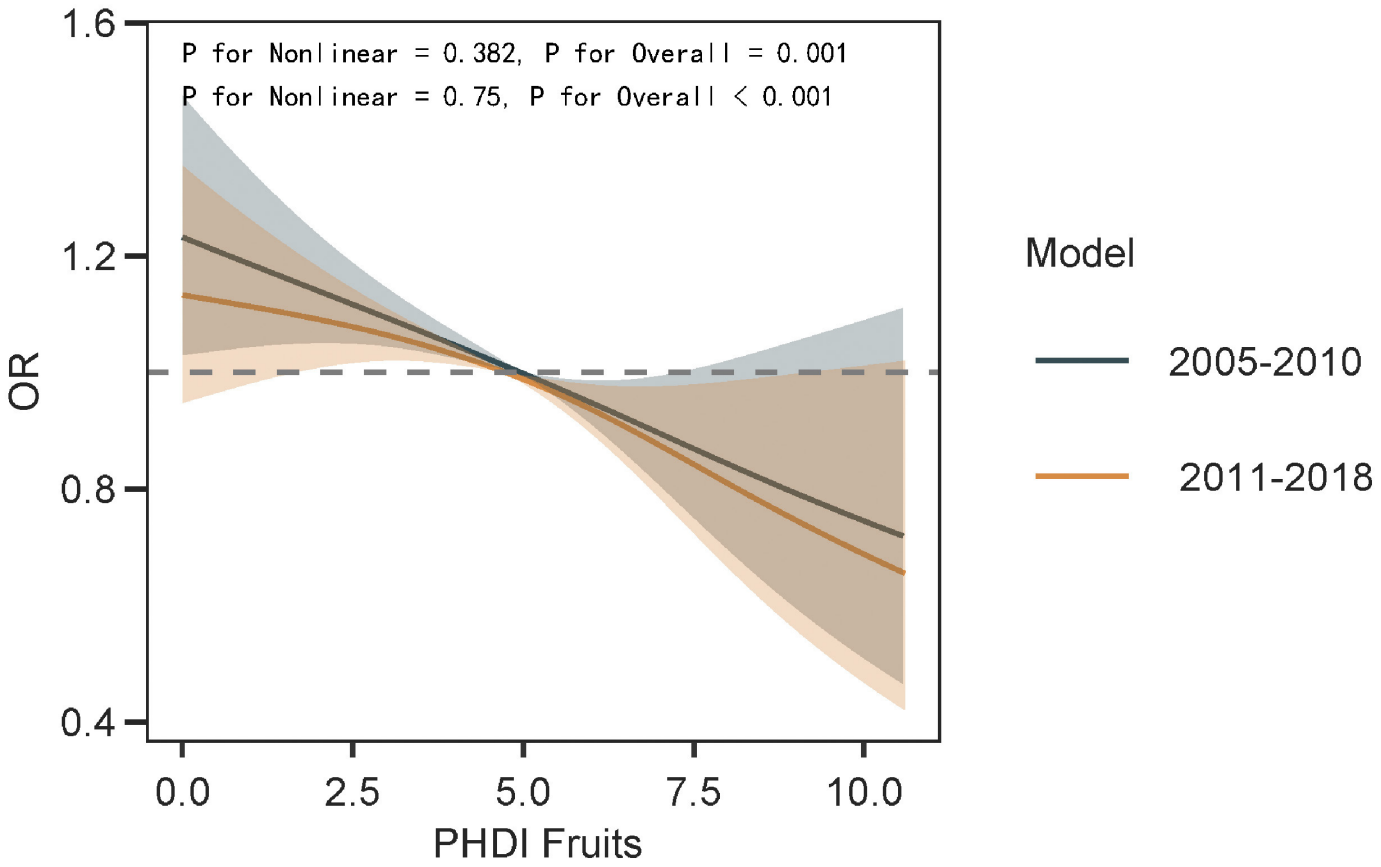
The impact of dietary cultural changes on the dose-response relationship between PHDI-Fruits and depression risk was examined using multivariable restricted cubic splines (RCS).

The association between PHDI-Fruits and depressive symptoms was then evaluated using multivariable logistic regression, as shown in Table 3. After full adjustment for covariates (Model 3), the highest quartile of PHDI-Fruits (Q4) exhibited a significantly stronger association with reduced odds of depressive symptoms compared to the original PHDI. Specifically, the OR for Q4 of PHDI-Fruits was 0.67 (95% CI: 0.58–0.78, *p* < 0.01), indicating a more robust protective effect against depressive symptoms compared to the original PHDI (Table 2), and comparable to or exceeding the effectiveness of indices such as AHEI and HEI. To further compare the predictive performance of the modified index, multivariable linear regression was performed to assess the relationship between the dietary indices and PHQ-9 scores (Table 4). Among all indices, PHDI-fruits had the largest negative coefficient (−0.0432, *p* < 0.01), indicating the strongest association with depressive symptom scores (Figure 5). The absolute negative coefficient of the original PHDI was small (−0.0055, *p* = 0.01), and the negative coefficient of other indices such as AHEI (−0.0145, *p* < 0.01) was stronger than that of PHDI, but much weaker than that of the adjusted PHDI-FRUITS. DII was positively correlated with PHQ-9 score (0.2360, *p* < 0.01), which was consistent with its pro-inflammatory characteristics. The results of the multivariable cubic spline analysis for gender-specific effects indicate that, at low adherence levels, women benefit more from following the PHD diet than men. However, at moderate to high adherence levels, the benefits for men and women are similar (Figure 6). The results of the multivariable restricted cubic splines (RCS) analysis show that, compared to the 2005–2010 cohort, the 2011–2018 cohort derived greater benefits at the same level of PHDI adherence (Figure 7).

**Table 3:** Association of PHDI-fruits with depression.

**Variables**	**Model 1 [OR (95%CI)]**	***p*-value**	**Model 2 [OR (95%CI)]**	***p*-value**	**Model 3 [OR (95%CI)]**	***p*-value**
Non-DEP	Reference	–	Reference	–	Reference	–
With DEP	0.92 (0.91–0.94)	< 0.01	0.93 (0.91–0.94)	< 0.01	0.95 (0.94–0.97)	< 0.01
**Interquartile**						
Quartile 1	Reference	–	Reference	–	Reference	–
Quartile 2	0.77 (0.68–0.88)	< 0.01	0.77 (0.68–0.88)	< 0.01	0.92 (0.80–1.04)	0.18
Quartile 3	0.61 (0.53–0.70)	< 0.01	0.62 (0.54–0.70)	< 0.01	0.81 (0.70–0.3)	< 0.01
Quartile 4	0.49 (0.43–0.57)	< 0.01	0.50 (0.43–0.58)	< 0.01	0.67 (0.58–0.78)	< 0.01
*p* for trend	–	< 0.01	–	< 0.01	–	< 0.01

Model 1, No covariates were adjusted; Model 2, Adjusted for gender and age; Model 3, Adjusted for age, gender, race, PIR, education, BMI, smoking, and hypertension.

DEP, Depression; OR, Odds Ratio; CI, Confidence Interval.

**Table 4:** Multiple linear regression analysis of the association between dietary indices and PHQ-9 scores.

**Variable name**	**Estimate**	**Std-Error**	***t*_value**	***p*-value**	**CI-2.5**	**CI-97.5**
(Intercept)	3.8116	0.2164	17.6169	0.0000	3.3875	4.2357
PHDI_US	−0.0055	0.0022	−2.5586	0.0105	−0.0097	−0.0013
PHDI_Fruits	−0.0432	0.0102	−4.2384	0.0000	−0.0632	−0.0232
AHEI	−0.0145	0.0040	−3.5727	0.0004	−0.0224	−0.0065
DII	0.2360	0.0214	11.0489	0.0000	0.1941	0.2779
HEI2020	−0.0034	0.0037	−0.9244	0.3553	−0.0106	0.0038
MEDI	0.0117	0.0345	0.3403	0.7336	−0.0559	0.0793

PHDI, Planetary Health Diet Index; AHEI, Alternative Healthy Eating Index; DII, Dietary Inflammatory Index; HEI, Healthy Eating Index; MEDI, Mediterranean Diet Index; CI, Confidence Interval.

### 4.4 Sensitivity analysis

The recalibrated PHDI-Fruits index demonstrated improved predictive performance for depressive symptoms compared to the original PHDI. The AUC increased from 0.57 (95% CI: 0.56–0.58) to 0.61 (95% CI: 0.60–0.63), indicating better discrimination. Sensitivity improved slightly (0.43 to 0.50), while specificity remained consistent (0.68). Notably, the negative predictive value (NPV) increased significantly from 0.40 to 0.83, suggesting that PHDI-Fruits more effectively identifies individuals without depressive symptoms (Table 5).

**Table 5:** Predictive performance of PHDI and PHDI-fruits for depression.

**Models**	**AUC (95% CI)**	**Sensitivity (95% CI)**	**Specificity (95% CI)**	**PPV (95% CI)**	**NPV (95% CI)**
PHDI	0.57 (0.56–0.58)	0.43 (0.42–0.56)	0.68 (0.55–0.70)	0.71 (0.69–0.72)	040 (0.39–0.41)
PHDI-Fruits	0.61 (0.60–0.63)	0.50 (0.35–0.53)	0.68 (0.67–0.83)	0.31 (0.29–0.36)	0.83 (0.82–0.84)

AUC, area under the receiver operating characteristic curve; CI, confidence interval; PPV, positive predictive value; NPV, negative predictive value.

## 5 Discussion

Since the EAT-Lancet Commission proposed the “Planetary Health Diet” (PHD) in 2019, its dual objectives of promoting health and environmental sustainability have garnered increasing global attention (10). While PHD has been widely recognized as a framework to enhance individual health and ecological sustainability, its applicability in the U.S. context and its association with specific health outcomes, such as mental health, remain underexplored (1). The PHDI is the first global dietary index that systematically integrates the dual dimensions of “human health” and “planetary health”. In contrast, HEI (which focuses on nutrient adequacy) and AHEI (which emphasizes chronic disease prevention) solely address the health dimension. Unlike traditional indices that are purely nutrition-oriented, the PHDI innovatively incorporates four environmental indicators: carbon footprint, blue water usage, land use, and biodiversity impact (10, 28). This study systematically analyzed the association between the U.S. Planetary Health Diet Index (PHDI-US) and depressive symptoms, comparing its performance with other dietary indices, including AHEI, HEI-2020, MEDI, and DII. Results demonstrated that higher PHDI-US adherence was significantly associated with reduced risk of depressive symptoms and was closely linked to the intake of core dietary components, particularly fruits, promoted by healthy dietary patterns. However, compared to other dietary indices, PHDI-US exhibited relatively weaker performance in mitigating depressive symptoms. After optimizing the weights of PHDI components through machine learning techniques, the recalibrated PHDI-Fruits showed significantly enhanced protective effects. These findings underscore the importance of optimizing dietary indices for specific health outcomes during their design and application. While PHDI is theoretically advantageous for its dual focus on health and sustainability, its practical impact on mental health requires further optimization and empirical validation to inform more precise dietary intervention strategies (29–32).

In recent years, the U.S. National Strategy on Hunger, Nutrition, and Health has provided crucial policy support for advancing sustainable dietary patterns. The strategy calls for enhanced collaboration among federal agencies to improve public access to healthy and sustainable diets while advocating for more comprehensive research to assess the complex relationships among dietary quality, mental health, and sustainability. However, research on how dietary indices simultaneously promote health, psychological wellbeing, and environmental sustainability remains limited. In particular, there is a lack of systematic frameworks that integrate diverse dietary indices, such as PHDI, AHEI, HEI-2020, and MEDI, with mental health and sustainability metrics. One major contribution of this study was the recalibration of PHDI-US to create a revised index, PHDI-Fruits, which incorporated weighted adjustments for fruit intake. Feature importance analysis using machine learning identified fruits as the most critical component among the 16 food groups in PHDI-US for reducing depressive symptoms. This finding aligns with prior research emphasizing the benefits of fruit consumption for mental health, which include their richness in vitamins, minerals, antioxidants, and fiber, as well as their potential to modulate gut microbiota and reduce systemic inflammation (33–35). The recalibrated PHDI-Fruits index demonstrated significantly superior predictive performance compared to the original PHDI-US, as evidenced by stronger associations with reduced odds of depressive symptoms and lower PHQ-9 scores. Sensitivity analyses further confirmed the enhanced effects of PHDI-Fruits, particularly its significant improvements in discrimination (AUC) and negative predictive value. To analyze the impact of changes in dietary culture on the results, we conducted subgroup analyses based on the NHANES survey cycles (2005–2010 and 2011–2018). The results showed that, in recent years, as dietary culture has evolved, the benefits of adhering to PHDI-Fruits have become more pronounced. This suggests that we should reduce the consumption of red meat and other similar foods while increasing the intake of green fruits and vegetables.

In terms of environmental sustainability, fruit production generally has a lower carbon footprint and water usage compared to other foods, such as red meat and highly processed foods. Increasing fruit intake can reduce environmental burdens, especially since the production of fruits exerts less pressure on ecosystems compared to high-energy-density animal products and processed foods. Furthermore, increasing the consumption of plant-based foods helps mitigate the negative environmental impacts of agriculture, such as greenhouse gas emissions, while promoting the sustainable use of soil and water resources. Therefore, advocating for increased fruit consumption not only benefits individual mental health but also contributes to environmental sustainability, thereby supporting global health and environmental protection efforts. Since the dietary data were based on a single 24-h dietary recall, this method may not fully capture an individual's long-term dietary patterns and could introduce recall bias. Although the 24-h dietary recall is widely used in large-scale population studies, its limitations should not be overlooked. This method may fail to accurately capture participants' long-term dietary patterns. Future research could consider using multiple dietary recalls or other more comprehensive dietary assessment methods to reduce recall bias. Furthermore, the absence of data on antidepressant use is a limitation, as it may confound the relationship between diet and depressive symptoms.

Although this study optimized the PHDI-Fruits, the optimization was validated only in the training dataset. Future research should validate its robustness using independent datasets to ensure its generalizability across different populations. The optimization of PHDI-Fruits was based on data from the U.S. population, but dietary cultural differences across regions may limit its applicability. For example, diets in Asia are more reliant on grains. Therefore, future research should consider regional adjustments to the index to accommodate different dietary cultures and population characteristics. The optimization of PHDI-Fruits was based on the random forest algorithm; however, its adjustment process can still be interpreted from a biological perspective. Fruits are rich in antioxidant components, such as vitamin C and flavonoids, which have been shown to reduce oxidative stress and inflammation levels—factors closely associated with the onset of depression (36, 37). Therefore, the weight adjustment of fruits in PHDI-Fruits is reasonable and supported by existing literature. While the PHDI-Fruits index shows promising results, it was developed and tested within the same dataset, and has not yet been externally validated. Therefore, its potential use as a preventive or clinical tool should be considered with caution until further validation studies are conducted. Future research should consider the differences in fruit quality, particularly between organic and non-organic fruits, as non-organic fruits may contain pesticide residues, which could influence their role in preventing depression. Existing studies suggest that pesticide residues may be associated with neurotoxic effects. Therefore, further research is needed to explore this potential threshold effect. Dietary supplements, such as vitamin D and fish oil, have been shown to have potential effects on depression. However, due to data limitations and issues with collinearity, this study was unable to adjust for these supplements as covariates. Future research should consider including these supplements to further refine the understanding of the relationship between diet and depression.

Our findings are consistent with previous studies, indicating that adherence to high-quality dietary patterns, such as the Healthy Eating Index (HEI), the Alternate Healthy Eating Index (AHEI), and the Mediterranean diet, is associated with a reduced risk of depressive symptoms (38, 39). The relationship between the Dietary Inflammatory Index (DII) and increased depression risk also aligns with previous research (40). Both HEI and AHEI emphasize the intake of fruits, vegetables, whole grains, and unsaturated fats, which are also core components of the PHDI (41–45). However, compared to earlier indices, PHDI offers a clear advantage by incorporating environmental sustainability indicators. More importantly, we provide evidence supporting the optimization of the PHDI, making it a potentially more suitable dietary approach for populations at high risk for depression. These findings suggest that recalibrating dietary indices to reflect the weighted contributions of key dietary components can improve their utility as tools for promoting mental health. The results highlight the need to prioritize dietary interventions that include high fruit intake and other nutrient-dense plant-based foods. Although the original PHDI-US framework was based on health and environmental sustainability, it underestimated the importance of fruits for mental health outcomes, underscoring the need for targeted adjustments to optimize its potential for depression prevention. The superiority of the recalibrated PHDI-Fruits highlights the importance of tailoring dietary frameworks for specific health outcomes. Dietary indices designed for chronic disease prevention or environmental sustainability may require recalibration to address mental health needs. This approach could guide future dietary recommendations that address the multidimensional aspects of physical, psychological, and environmental wellbeing. Additionally, this study highlights the practicality of integrating machine learning methods, such as random forest regression, into dietary research. These methods facilitate the identification of key dietary determinants of health outcomes, providing valuable insights for optimizing and improving dietary indices. In large, complex datasets, such methods may be more effective than traditional statistical approaches in capturing subtle relationships (32, 46, 47).

The strengths of this study include the use of a nationally representative sample from NHANES, rigorous dietary assessment methods, and advanced statistical and machine learning techniques to enhance the reliability of the findings. However, there are several limitations before these results can be applied in clinical practice. While this study identifies a significant association between PHDI and depression, its cross-sectional design limits the ability to infer causality. Therefore, future longitudinal studies would be valuable in elucidating whether dietary patterns exert a temporal influence on the onset or alleviation of depression. While PHQ-9 is a validated tool for assessing depressive symptoms, it relies on self-reported data, which may be subject to recall bias. The recalibration of PHDI-US primarily focused on fruit intake, but other dietary components and numerous potential confounders may also play a critical role in mental health outcomes. Despite comprehensive adjustment for potential confounders, the possibility of residual confounding cannot be ruled out. Future research should explore the combined effects and interactions of multiple dietary components and confounders.

## 6 Conclusion

This study highlights the association between adherence to PHDI-US and reduced depressive symptoms. By emphasizing the critical role of fruits, the recalibrated PHDI-Fruits outperformed the original PHDI-US and other established dietary indices in reducing depressive symptoms, demonstrating its potential as a tailored dietary intervention tool for mental health promotion. These findings underscore the importance of optimizing dietary indices for specific health outcomes and provide a framework for future research and public health initiatives aimed at integrating diet and mental health.

## Data Availability

The datasets presented in this study can be found in online repositories. The names of the repository/repositories and accession number(s) can be found below: https://www.cdc.gov/nchs/nhanes/index.html.
